# Fyn Knock-Down Prevents Levodopa-Induced Dyskinesia in a Mouse Model of Parkinson’s Disease

**DOI:** 10.1523/ENEURO.0559-20.2021

**Published:** 2021-07-12

**Authors:** Melina P. Bordone, Ana Damianich, Alejandra Bernardi, Tomas Eidelman, Sara Sanz-Blasco, Oscar S. Gershanik, M. Elena Avale, Juan E. Ferrario

**Affiliations:** 1 Facultad de Ciencias Exactas y Naturales, Instituto de Biociencias, Biotecnología y Biología traslacional (iB3), Universidad de Buenos Aires, Ciudad Autónoma de Buenos Aires C1428EGA, Argentina; 2 Consejo Nacional de Investigaciones Científicas y Técnicas (CONICET), Ciudad Autónoma de Buenos Aires C1113AAD, Argentina; 3 Instituto de Investigaciones en Ingeniería Genética y Biología Molecular (INGEBI) Dr. Héctor N. Torres, (CONICET), Ciudad Autónoma de Buenos Aires C1428ADN, Argentina; 4 Facultad de Farmacia y Bioquímica, Instituto de Investigaciones Farmacológicas (ININFA), Universidad de Buenos Aires, Ciudad Autónoma de Buenos Aires C1113AAD, Argentina

**Keywords:** Fyn, levodopa-induced dyskinesias, microRNA, NMDA, Parkinson’s disease, RNA therapy

## Abstract

Dopamine replacement by levodopa (L-DOPA) is the most widely used therapy for Parkinson’s disease (PD), however patients often develop side effects, known as L-DOPA-induced dyskinesia (LID), that usually need therapeutic intervention. There are no suitable therapeutic options for LID, except for the use of the NMDA receptor (NMDA-R) antagonist amantadine, which has limited efficacy. The NMDA-R is indeed the most plausible target to manage LID in PD and recently the kinase Fyn, one of its key regulators, became a new putative molecular target involved in LID. The aim of this work was to reduce Fyn expression to alleviate LID in a mouse model of PD. We performed intrastriatal delivery of a designed micro-RNA against Fyn (miRNA-Fyn) in 6-OHDA-lesioned mice treated with L-DOPA. The miRNA-Fyn was delivered either before or after L-DOPA exposure to assess its ability to prevent or revert dyskinesia. Preadministration of miRNA-Fyn reduced LID with a concomitant reduction of FosB-ΔFosB protein levels, a marker of LID, as well as decreased phosphorylation of the NR2B-NMDA subunit, which is a main target of Fyn. On the other hand, post-L-DOPA delivery of miRNA-Fyn was less effective to revert already established dyskinesia, suggesting that early blocking of Fyn activity might be a more efficient therapeutic approach. Together, our results provide proof of concept about Fyn as a plausible therapeutic target to manage LID, and validate RNA silencing as a potential approach to locally reduce striatal Fyn, rising new perspectives for RNA therapy interventions in PD.

## Visual Abstract

**Figure:**
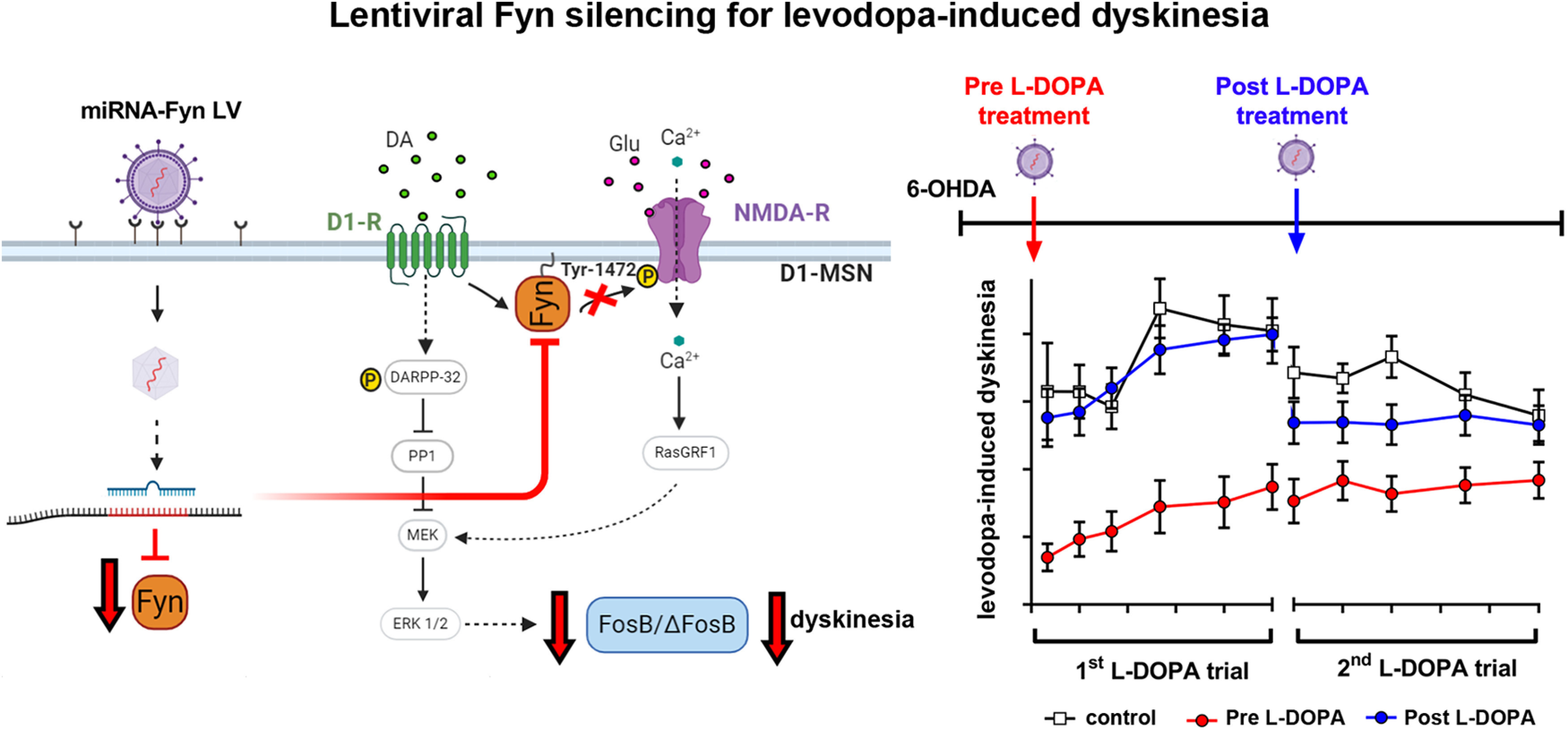

## Significance Statement

Levodopa (L-DOPA)-induced dyskinesia (LID) is an incapacitant side effect of treatment in Parkinson’s disease (PD). LID is a therapeutic challenge, lacking an effective pharmacological treatment, except for the use of inhibitors of the NMDA receptor (NMDA-R), which have limited efficacy and may trigger untoward side effects. The kinase Fyn is a key regulator of NMDA function and a potential therapeutic target to control LID. Here, we show that RNA interference therapy to reduce the amount of Fyn mRNA in the adult brain is effective to prevent LID in a mouse model of PD, setting the grounds for future biomedical interventions to manage LID in PD.

## Introduction

Parkinson’s disease (PD) is a highly prevalent neurodegenerative disorder with only symptomatic treatments available. Levodopa (L-DOPA) provides significant benefit in PD patients, however, its prolonged administration leads to disabling side effects known as L-DOPA-induced dyskinesia (LID; Obeso et al., 2017; Espay et al., 2018). As almost 90% of patients develop LID after 10 years of treatment, its management remains one of the greatest challenges in PD research.

Currently, amantadine is the only available drug proved to be clinically useful to reduce LID. Its antidyskinetic effects are attributed to the non-competitive antagonism of the NMDA receptor (NMDA-R) subtype, supporting the hypothesis that NMDA-R activity is critical for the development of LID (Bastide et al., 2015; Espay et al., 2018). However, long-term use of amantadine may trigger undesired side effects such as cognitive impairment, hallucinations, nausea and edema, among others (Thomas et al., 2004; Sawada et al., 2010; Perez-Lloret and Rascol, 2018). For several patients, the only alternative is to reduce the dose of L-DOPA, with the concomitant reduction of its antiparkinsonian effect.

The analysis of molecular pathways underlying LID has been crucial to identify novel potential targets for pharmacological intervention (Heumann et al., 2014). Striatonigral medium spiny neurons of the direct pathway (dMSNs), which express dopamine D1 receptor (D1-R), have a critical role in LID (Darmopil et al., 2009; Bastide et al., 2014). Stimulation of D1-R activates the canonical dopaminergic signaling pathway, but also, cross talks with the NMDA-R (Dunah et al., 2004; Trepanier et al., 2012), which in turn triggers excitatory postsynaptic potentials and calcium-mediated intracellular signaling (Heumann et al., 2014; Bastide et al., 2015). Indeed, drugs used to modulate NMDA-R, such as the antagonist CP-101606 have been tested as therapeutic approaches for LID, but the appearance of side effects under long-term treatment led to their discontinuation (Nash et al., 2004; Nutt et al., 2008; Kong et al., 2015). In this scenario, a myriad of endogenous modulators of the NMDA-R activity could be explored as potential targets to tackle LID.

NMDA-R and D1-R cross talk at the postsynaptic density zone, involving PSD-95 (Porras et al., 2012) and Fyn, a member of the Src family tyrosine kinases (SFK). Fyn phosphorylates the NR2B subunit of NMDA-R at Y-1472 (Nakazawa et al., 2001). This phosphorylation disrupts NR2B binding to the adaptor protein 2 complex and suppresses clathrin-mediated endocytosis of the NMDA-R (Lavezzari et al., 2003). Thus, Fyn activity stabilizes NMDA-R to the membrane (Nakazawa et al., 2001), increasing the neuronal response to glutamate. We have proposed that a reduction in Y-1472 phosphorylation would reduce NMDA-R activity and consequently, decrease LID (Sanz-Blasco et al., 2018). Previous work from our group demonstrated that Fyn deficient mice develop less LID and accumulate less FosB-ΔFosB in the striatum than wild type. Moreover, saracatinib (AZD0530), a non-selective pharmacological inhibitor of Fyn, prevented the development of dyskinesia (Sanz-Blasco et al., 2018).

Classical pharmacology, extremely powerful for some targets and purposes, is sometimes limited for widely expressed molecules, making necessary the development of precision pharmacology based in novel technologies such as gene and mRNA therapies. Therefore, we sought to achieve a localized reduction of Fyn into striatal neurons of dyskinetic mice, taking advantage of RNA silencing tools.

In the work presented herein, we validated Fyn as a therapeutic target against LID and developed an RNA therapy strategy based on Fyn down-regulation in a preclinical mouse model of LID. We show that Fyn silencing prevents the development of LID and may also revert already established dyskinesia. Our results highlight the role of Fyn in the development of LID and support the use of miRNA-Fyn as a novel tool with potential translational value as a therapeutic option for the management of LID.

## Materials and Methods

### miRNA-Fyn design and cloning

miRNA-Fyn molecules were designed to target the *Mus musculus Fyn* protooncogene mRNA (Gene Bank: BC092217.1) using a siRNA selection algorithm, freely available at http://sirna.wi.mit.edu/home.php (Yuan et al., 2004). Four siRNAs sequences were selected at positions +442 (GGGCTGTGTGCAATGTAAGGA), +691 (GACACTGTTTGTGGCGCTTTA), +1007 (CGTGATTGGGATGATATGAAA), and +1134 (CAGAGAAAGCTGATGGTTTGT). miRNAs were designed based on the backbone of the mouse miRNA-155. Sequences containing each miRNA were synthetized by GenScript and cloned into the pUC57 plasmid. miRNA cassettes were then subcloned into a third generation lentiviral vector (LV), under the human synapsin neuronal promoter, between the AgeI and EcoRI cloning sites of a pTrip LV backbone, as previously reported (Espíndola et al., 2018). The miRNA construct we produced does not have a reporter gene, as it might interfere with miRNA processing. The control vector carries a non-silencing sequence in the same backbone. Lentiviral particles were generated as described previously (Avale et al., 2013; Espíndola et al., 2018). miRNA-Fyn-LV silencing vector is available from Addgene.

### Animals and surgery

The study was performed on C57BL6J-FCEN female mice (an inbreed strain from Facultad de Ciencias Exactas y Naturales, Universidad de Buenos Aires), from three to six months old, weighing 25–30 g at the beginning of the experiments. All surgical procedures and experimental manipulations were performed in accordance with the European Directive 2010/63/EU and approved by the Ethics Committee of Facultad de Farmacia y Bioquímica (Universidad de Buenos Aires).

The mouse model of PD was generated as previously detailed (Sanz-Blasco et al., 2018). Briefly, mice were anesthetized with isoflurane 0.5–2% (Baxter) in medical grade oxygen with an air flow at 2.5 l min^−1^ and placed into a stereotaxic frame (Stoelting Co). The dopaminergic pathway was unilaterally lesioned by injecting 1 μl of 6-OHDA (Sigma) at 3.4 μg/μl, free-base diluted in 0.1% ascorbic acid and a rate of 0.5 μl/min into the left medial forebrain bundle (MFB) at coordinates: AP: −0.1 mm, ML: +0.11 mm, DV: −0.5 mm from bregma, ensuring that bregma and λ were at the same horizontal point, according to the *Mouse Brain Atlas* (Paxinos and Franklin, 2001). Mice received intense nursing care during the ensuing three weeks to reduce weight loss, avoid suffering, and increase survival rates. Twice daily, mice received rehydration therapy (up to 1 ml of 5% dextrose in water, s.c.) and wet mash diet according to individual needs.

Wet pellets were supplemented with sunflower-raisins, multicereal preparation (Nestum, Nestle), and yeast gelatin preparation. This diet started one week prior to surgery to adapt the animals to the taste of this food supplement and to improve both their weight and physical condition. After surgery, mice were kept at warmer temperature (23–26°C) and weighed every 2 d. Three weeks after 6-OHDA lesion, mice were tested in the cylinder test to evaluate motor impairment because of dopaminergic degeneration (Lundblad et al., 2004). Briefly, rodents were placed in a plastic transparent cylinder (diameter × height: 10 × 20 cm), and the number of times they stood on their rear paws were counted. Mice welfare was considered throughout the study, and human endpoint after surgery was required only for one mouse.

### Experimental design

From a total of 76 mice lesioned with 6-OHDA to develop the PD model, only those mice with evidence of severe DA loss (spontaneous ipsilateral rotation and use of ipsilateral paw >85% times in the cylinder test) were included in the study. This selection led to 59 mice that were assigned to the two treatment trials; 29 for the pre-L-DOPA treatment and 30 for the post-L-DOPA treatment. Within each trial, mice were randomly assigned to three experimental groups (no LV; control LV or miRNA-Fyn LV). For the pre-L-DOPA treatment, four to five weeks after recovery from the 6-OHDA lesion, mice were stereotaxically injected into the striatum either with control LV (*n* = 6), miRNA-Fyn LV (*n* = 17), or without LV (*n* = 6). We left five to six weeks after the lentiviral injection to allow expression of the miRNA, then all mice were challenged with L-DOPA for two weeks to induce dyskinesia (see details below). For the post-L-DOPA treatment group, a total of 30 6-OHDA-lesioned mice were first treated with L-DOPA for two weeks, assessed for LID, and then randomly assigned to receive either control LV (*n* = 5), miRNA-Fyn LV (*n* = 19), or nothing (*n* = 6). After four weeks, all mice were re-challenged with L-DOPA.

**Figure 1.**
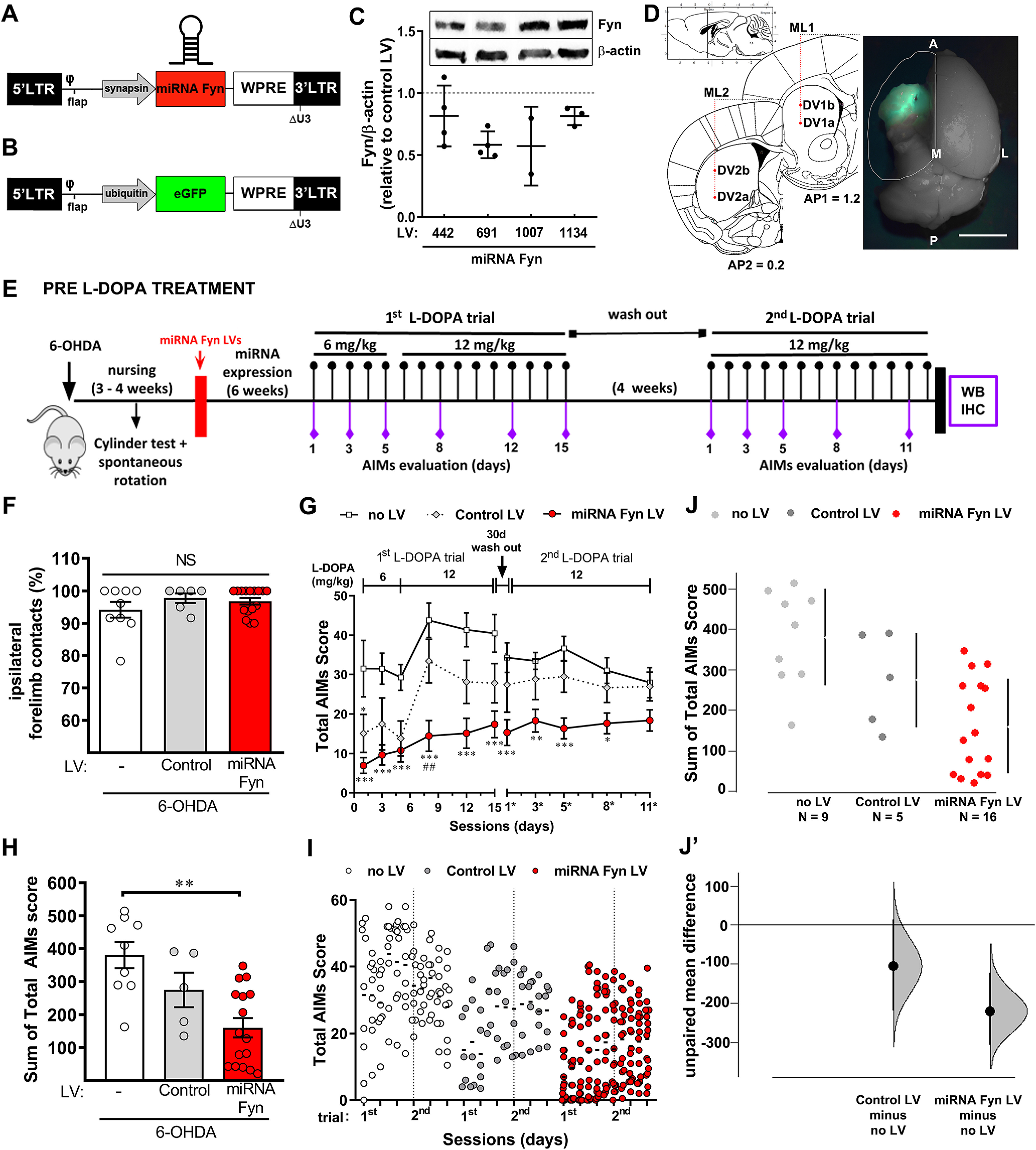
miRNA strategy to reduce striatal Fyn as a preventive treatment for LID. ***A***, ***B***, Schema of LVs used to express the miRNA (miRNA) to target Fyn (***A***) or the reporter gene GFP (***B***), used to set the coordinates for the intrastriatal injections. ***C***, Western blot quantification of Fyn/β-actin protein levels in mouse N2a cell-line treated with each miRNA LV. Data are expressed relative to control-LV-treated cells. We selected the miRNA-Fyn 691 because it reduced Fyn amounts in four independent experiments. ***D***, Coronal schemes depicting the injection sites used to deliver LVs into the striatum. Overview of striatal expression of the reporter GFP. Scale bar: 5 mm. A: anterior; P: posterior; M: medial; L: lateral, AP: antero-posterior; ML: medio-lateral; DV: dorso-ventral. ***E***, Timeline of the preventive paradigm used: 6-OHDA lesion into the MFB, striatal miRNA LV injection, L-DOPA administration and behavioral analyses followed by postmortem analysis. ***F***, Cylinder test performed after 6-OHDA lesion, prior to random assignment of mice to treatment groups. Data are mean ± SEM. Kruskal–Wallis test *H*_(2)_ = 1.211; *p* = 0.5457. ***G***, Sum of values for axial dystonia, orolingual and limb dyskinesia (total AIMs score) per day throughout the L-DOPA treatment. Experimental groups: non-injected (no LV; *n* = 9), injected with control LV (*n* = 5), or with miRNA-Fyn LV (*n* = 16). Data are mean ± SEM. Two-way ANOVA with repeated measures (interaction: *F*_(20,270)_ = 3.440, *p* < 0.0001; time: *F*_(10,270)_ = 13.08, *p* < 0.0001; treatment: *F*_(2, 27)_ = 10.32, *p* = 0.0005; subject: *F*_(27,270)_ = 35.33, *p* < 0.0001) and *post hoc* Tukey’s test; *p < 0.05, **p < 0.01, and ***p < 0.001 versus no LV; ## versus control LV. ***H***, Sum of total AIMs score from all sessions. Data are mean ± SEM. Kruskal–Wallis test (*H*_(2)_ = 12.52; *p* = 0.0019) followed by Dunn’s test (*p* = 0.0014, no LV vs miRNA-Fyn LV). ***I***, Dot plot of data showing the distribution of AIMs score within each group. The dotted line separates the first from the second L-DOPA trial. ***J***, ***J’***, Sum of total AIMs score analyzed by estimation statistic shown as a Cumming estimation plot. On the upper axes (***J***), the raw data are presented as a swarmplot and mean ± SD are represented on the right of each experimental group as a gap between the vertical lines and vertical lines, respectively. On the lower axes (***J’***), the unpaired mean difference for two comparisons against the shared control (no LV group) are shown. The unpaired mean differences (M_diff_) are plotted as bootstrap sampling distributions. Each mean difference is depicted as a dot. Each 95% CI is indicated by the ends of the vertical error bars. Unpaired M_diff_ (control LV vs no LV) = −106 and 95.0% CI [−234.0, 5.7] with a *p* = 0.134 for the two-sided permutation *t* test. Unpaired M_diff_ (miRNA-Fyn LV vs no LV) = −220 and 95.0% CI [−300, −112] with a *p* = 0.0006 for the two-sided permutation *t* test. *Figure Contributions*: M. Elena Avale and Juan E. Ferrario designed miRNA-Fyn sequences. Melina P. Bordone cloned the vectors. M. Elena Avale and Ana Damianich prepared LVs particles. M. Alejandra Bernardi and Melina P. Bordone performed *in vitro* experiments and Western blot detection. Ana Damianich and Juan E. Ferrario set the striatal injection sites. Melina P. Bordone performed 6-OHDA lesions and Ana Damianich striatal lentiviral injections. Melina P. Bordone, Ana Damianich, Tomas Eidelman, and Juan E. Ferrario made animal nursing and cylinder tests. Melina P. Bordone, Tomas Eidelman, and Sara Sanz-Blasco scored LIDs. Melina P. Bordone performed statistical analyses and Melina P. Bordone, Juan E. Ferrario, and M. Elena Avale analyzed and discussed data.

The “no LV” groups of mice were not injected with LV but received the same schedule of L-DOPA administration either in the pre-L-DOPA or post-L-DOPA trial and run in parallel to the other groups.

Five mice from the pre-L-DOPA trial died or were sacrificed for humanitarian reasons and are not included in the analysis (one from the miRNA-Fyn, one from the control LV and three from the no-LV group). Some samples used for biochemical analyses (pNR2B) were missed because of technical issues or finished in previous determinations, but we always performed experiments with the total available material.

### Lentiviral injections

Mice were anesthetized as above and set into the stereotaxic frame. 1.5 μl of lentiviral suspension (0.2 × 10^7^ TU/ml) were injected per site at 0.2 μl/min into the left striatum, through a 36-G stainless steel needle coupled to a 10-μl Hamilton syringe, at four injection sites at the following coordinates (from bregma): AP_1_: +1.2 mm, ML_1_: +1.7 mm, DV_1a_: –3.8 mm; DV_1b_: –3 mm; AP_2_: +0.2 mm, ML_2_: +2.3 mm, DV_2a_: –4 mm, and DV_2b_: –3 mm. (Paxinos and Franklin, 2001). These coordinates were set up previously using an EGFP-reporter LV that bears the same envelope and backbone than miRNA LVs and infect similar area that the experimental ones (Fig. 1*D*).

### Pharmacological treatments and behavior

Dyskinesia were induced with L-DOPA/benserazide (Sigma, catalog #D9628/B7283) as before (Sanz-Blasco et al., 2018). Abnormal involuntary movements (AIMs) were evaluated by a blinded operator following standard protocols (Lundblad et al., 2005; Cenci and Lundblad, 2007) and as reported (Sanz-Blasco et al., 2018) on days 1, 3, 5, 8, 12, and 15 for the first L-DOPA trial, and 1, 3, 5, 8, and 11 for the second one. On each testing day, animals were observed and scored for 1 min at 20-min intervals until no further AIMs were detectable. Three categories of AIMs were observed. (1) Orofacial: discrete vertical (open and close) jaw movements towards the contralateral side, with eventual tongue protrusion. (2) Forelimb: twitching or jerking movements of the forelimb contralateral to the lesion of a choreic (non-rhythmic, spasmodic) or ballistic (choreic movements of a larger amplitude) pattern. (3) Dystonic: lateral deviation of the trunk, neck, and head toward the contralateral side, leading to a loss of orthostatic equilibrium. The frequency and intensity of each AIM were evaluated using a standard scale: 0 = absent; 1 = present for less than half of the observation period; 2 = present for more than half of the observation period; 3 = present constantly but suspended by a sensorial stimulus; 4 = present constantly, irrespective of a stimulus. Values for each category were pooled per animal for each day, obtaining the mean per day for each experimental group.

### Postmortem analysis

One hour after the last L-DOPA injection, mice were sacrificed by cervical dislocation and brains immediately removed and coronally cut at the level of the optic chiasma. Both striata were dissected and rapidly frozen for Western blot analysis, while the caudal sections containing the midbrain were fixed by immersion in 4% paraformaldehyde (Biopack; catalog #959408) for 24 h for immunohistochemical detection of dopaminergic denervation. Tyrosine hydroxylase (TH) was immunostained at the substantia nigra pars compacta (SNpc) in free-floating coronal sections (Sanz-Blasco et al., 2018). The number of TH positive (+) cells in the SNpc was counted from coronal sections (–2.92 to –3.80 mm from bregma; Paxinos and Franklin, 2001) using the Mercator Navigator software (Exploranova) connected to a Nikon Eclipse 50i microscope.

Western blot analyses were performed as previously described (Sanz-Blasco et al., 2018). Antibodies and dilutions used were as follows: anti-Fyn (1:1000; Santa Cruz Biotechnology; catalog #sc-16 RRID:AB_631528), anti-β-actin (1:2000; Cell Signaling; catalog #8457L, RRID:AB_10950489), anti-pNR2B-Tyr1472 (1:400; Pel-Freez Biologicals; catalog #P43301-0, RRID:AB_476111), anti-NR2B (1:400; UC Davis/NIH NeuroMab Facility; catalog #75-097, RRID:AB_10673405), anti-TH (1:1000; Pel-Freez Biologicals; catalog #P40101 RRID:AB_2313713), anti-FosB-ΔFosB (1:500; Santa Cruz Biotechnology; catalog #sc-48 RRID:AB_631515), donkey anti-rabbit HRP-conjugate (1:2000; Thermo Fisher Scientific; catalog #A16035, RRID:AB_2534709), donkey anti-mouse HRP-conjugate (1:2000; Thermo Fisher Scientific; catalog #A16017, RRID:AB_2534691), and goat anti-rabbit Biotin-conjugate (1:1000; Thermo Fisher Scientific; catalog #A16108, RRID:AB_2534780).

### Statistical analysis

When appropriate data were analyzed using both the null-hypothesis significance testing and the novel estimation statistic approach (Calin-Jageman and Cumming, 2019; https://thenewstatistics.com/itns/) to reinforce the results. In the case of hypothesis testing, data are presented as mean ± SEM. Differences between biological conditions were determined using one or two-tailed unpaired Student’s *t* test, and one-way or two-way ANOVA with repeated measures followed by Tukey’s test for *post hoc* comparisons. Data that did not follow a Gaussian distribution (assayed by D’Agostino–Pearson omnibus (K2) normality test) were analyzed with the nonparametric Kruskal–Wallis test and if there was statistical significance between the groups it was followed by Dunn’s multiple comparisons test.

For estimation statistics, raw data were entered in https://www.estimationstats.com/ and performed a shared control analysis to get the results based on confidence intervals (CIs) and we used the Dabestr v.0.3.0 package for R (Ho et al., 2019) to generate a shared control Cumming plot and manage to change plot aesthetics (i.e., colors, dot sizes). In this graph raw data were plotted on the upper part and summary measurements (mean ± SD) are represented on the right of each experimental group, where the gap between the vertical lines (representing the SD) is the mean for each group. On the lower part, each mean difference is plotted as a bootstrap sampling distribution. Five thousand bootstrap samples were taken; the CI was bias-corrected and accelerated. Mean differences are depicted as dots; 95% CIs are indicated by the ends of the vertical error bars. To measure the effect size, we used the unpaired mean difference. We also provide *p* value(s) as the likelihood(s) of observing the effect size(s), if the null hypothesis of zero difference is true, using the permutation *t* test.

Also, we performed a principal component analysis (PCA) followed by hierarchical clustering on principal components (HCPC) to analyze mice after miRNA-Fyn LV treatment. The R package FactoMineR was used for multivariate analysis (Lê et al., 2008) and the missMDA package (Josse and Husson, 2016) was used to estimate three (out of 35) pNR2B/NR2B/β-actin missing values. Significance was set at *p* < 0.05 in all cases.

GraphPad Prism 8.01 for Windows (GraphPad Software) was used for plots and hypothesis statistical analysis, and R 4.0 (http://www.R-project.org/) with RStudio 1.4.1106 for estimation plots or with RStudio 1.2.5033 for PCA and HCPC (http://www.rstudio.com/).

## Results

### Validation of LV miRNA-Fyn sequences and intrastriatal lentiviral injections

We designed four miRNAs against *Fyn* that were cloned into the miRNA-155 backbone and packed into LVs (Fig. 1*A*). These LVs were primarily tested in mouse cultured neuronal cells (N2a) and we found that miRNA-Fyn 691 (from now on “miRNA-Fyn”) showed a reproducible knock-down effect of ∼50% of Fyn protein, observed in three independent experiments (Fig. 1*C*). Therefore, this construct was selected for the following *in vivo* studies. Because the striatum is a wide non-compartmentalized nucleus, to maximize the expression of the construct throughout this structure, we previously set up the stereotaxic coordinates using a LV that expresses the reporter gene EGFP (Fig. 1*B*). An injection schedule into four sites on the ipsilateral striatum (see methods) was chosen to achieve a widespread and consistent viral transduction (Fig. 1*D*), coordinates were selected to favor transduction in the dorsolateral striatum, which is principally involved in LID (Bastide et al., 2015).

Next, the effect of this miRNA-Fyn, to reduce dyskinesia, was determined in two experimental paradigms aimed to analyze either the preventive (pre-L-DOPA) or the restorative (post-L-DOPA) effects of Fyn silencing on LID expression.

### Pre-L-DOPA treatment schedule

#### AIMs

We first tested the preventive role of miRNA-Fyn in a pre-L-DOPA treatment paradigm (Fig. 1*E*). All mice were unilaterally injected with 6-OHDA into the MFB, and three to four weeks later, dopaminergic denervation was verified by the cylinder test (Fig. 1*F*) and spontaneous rotation (not reported). Successfully lesioned mice were randomly assigned to three experimental groups: two groups received either miRNA-Fyn LV or the control LV while the third one received no treatment (no LV). Five to six weeks after LV injection, mice received L-DOPA during 15 d (first L-DOPA trial) and were scored for AIMs including axial dystonia, forelimb dyskinesia and orofacial dyskinesia. Because each type of AIM showed similar profile during each session, data are presented as the sum of all AIMs. After four weeks wash-out, mice were treated again with L-DOPA for 11 d (second L-DOPA trial).

Dyskinetic behavior is very variable both in humans and animal models, showing wide data dispersion, often masking experimental evidence. This results in the use of a large number of animals, in order to get statistically significance, or discard not evident differences that may appear in a deep analysis. In view of this, we sought for different strategies to analyze data and support conclusions. We used estimation statistical analysis based on CIs as a complement of the more frequently used null hypothesis significance testing. To analyze behavioral data by estimation statistic, for the pre-L-DOPA treatment we generated for each mouse a unique value for LID by the sum of the AIMs score in each session day. This value is representative of the global intensity of LID throughout the experiment, and it is sometimes used alternatively (Bido et al., 2011; Feyder et al., 2016). In the first and second L-DOPA trials, we observed that total AIMs score was statistically reduced in the miRNA-Fyn-injected group compared with non-injected control mice, along the whole schedule, (Fig. 1*G*,*H*). We then estimated the CI for such samples and observed that the mean difference (M_diff_) between control no-LV and miRNA-Fyn was −220 [95.0%CI −300, −110], with a *p* value of two-sided permutation *t* test of 0.0006 (Fig. 1*J*,*J’*). This dual analysis strongly supports a physiological effect of the miRNA-Fyn in dyskinetic behavior.

**Figure 2.**
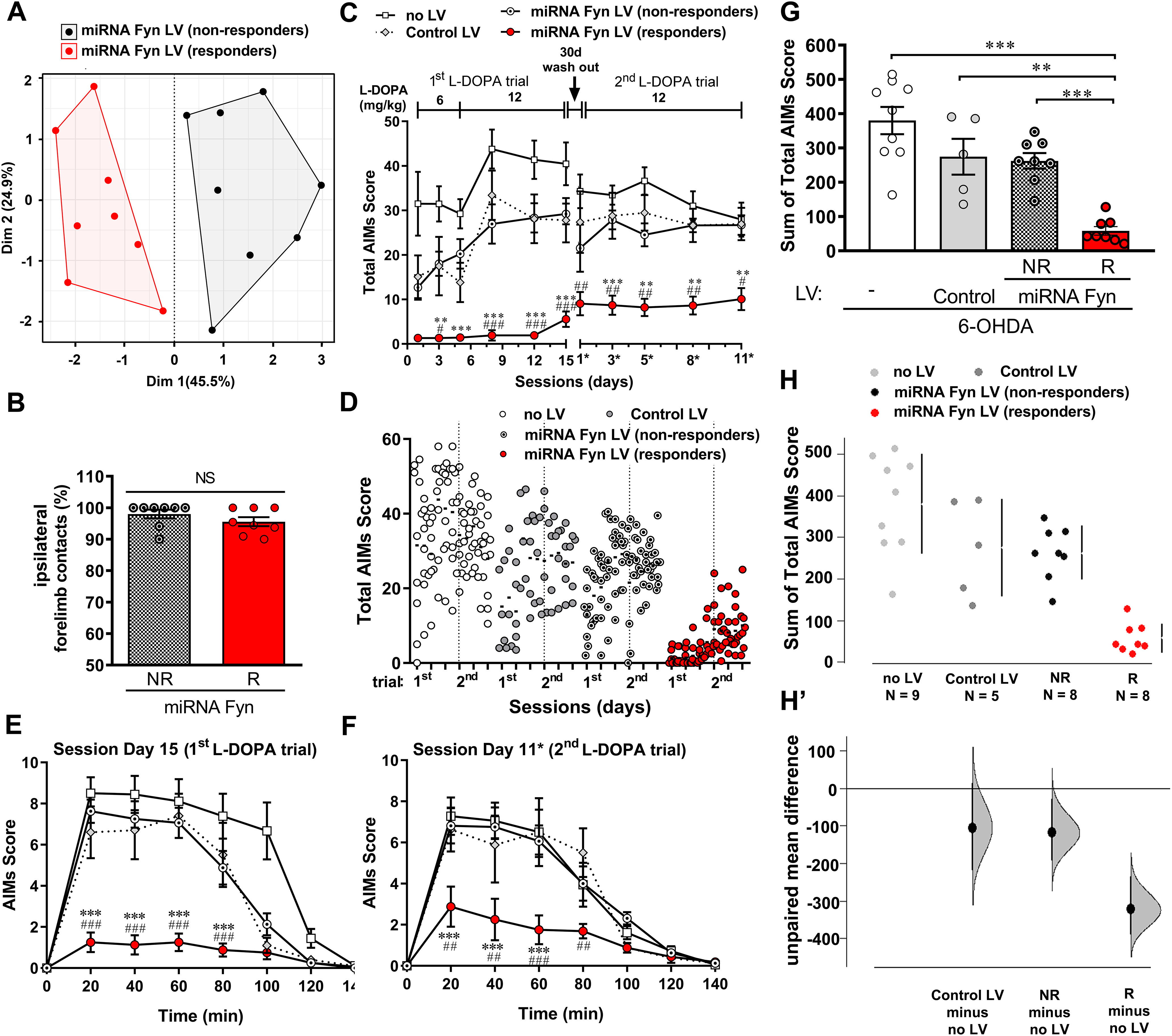
miRNA-Fyn treatment prevents the development of LID. ***A***, Factor map after HCPC split miRNA Fyn-injected mice into two groups assigned as R (*n* = 8) and NR (*n* = 8). ***B***, Cylinder test from miRNA Fyn-treated mice split into R and NR. Data are mean ± SEM. Two-tailed unpaired Student’s *t* test (*t* = 1.236, df = 14, *p* = 0.2367). ***C***, Total AIMs scores (as Fig. 1*G*) after discriminating R and NR miRNA-Fyn mice. Data are mean ± SEM. Two-way ANOVA with repeated measures (interaction: *F*_(30,260)_ = 3.361, *p* < 0.0001; time: *F*_(10,260)_ = 14.12, *p* < 0.0001; treatment: *F*_(3,26)_ = 18.96, *p* < 0.0001; subject: *F*_(26,260)_ = 21.63, *p* < 0.0001) and *post hoc* Tukey’s test; **p < 0.01, ***p < 0.001 versus NR; #*p* < 0.05, ##*p* < 0.01, and ###*p* < 0.001 versus control LV. ***D***, Dot plot of data showing the distribution of AIMs score after clustering miRNA-Fyn LV mice into R and NR groups. Dotted line separates the first and second L-DOPA trial. ***E***, ***F***, AIM score for session day 15 (from the first trial with L-DOPA; ***E***) and day 11* (from the second trial with L-DOPA; ***F***). Data are mean ± SEM. ***E***, Two-way ANOVA with repeated measures (interaction: *F*_(21,182)_ = 10.21, *p* < 0.0001; time: *F*_(7,182)_ = 97.39, *p* < 0.0001; treatment: *F*_(3,26)_ = 17.01, *p* < 0.0001; subject: *F*_(26,182)_ = 6.068, *p* < 0.0001). ***F***, Two-way ANOVA with repeated measures (interaction: *F*_(21,175)_ = 3.522, *p* < 0.0001; time: *F*_(7,175)_ = 64.41, *p* < 0.0001; treatment: *F*_(3,25)_ = 7.497, *p =* 0.0010; subject: *F*_(25,175)_ = 3.394, *p* < 0.0001) both followed by *post hoc* Tukey’s test; ***p < 0.001 versus NR; ##*p* < 0.01 and ###*p* < 0.001 versus control LV. ***G***, Sum of total AIMs score from all sessions (as Fig. 1*H*) after discriminating R and NR mice. Data are mean ± SEM. One-way ANOVA (*F*_(3,26)_ = 18.96, *p* < 0.0001) and *post hoc* Tukey’s test (*p* < 0.0001, *p* = 0.0013 and *p* = 0.005 for R vs no LV, control LV and NR, respectively). ***H***, ***H’***, Sum of total AIMs score analyzed by estimation statistic shown as a Cumming estimation plot (as Fig. 1*J*,*J’*) after discriminating R and NR mice. ***H***, The raw data are presented as a swarmplot and mean ± SD are represented on the right of each experimental group. ***H’***, Unpaired mean difference for three comparisons against the shared control (no LV group). The unpaired M_diff_ are plotted as bootstrap sampling distributions. Each mean difference is depicted as a dot and the 95% CI is indicated by the ends of the vertical error bars. Unpaired M_diff_ (control LV vs no LV) = −106 and 95.0% CI [−234.0, 5.7], unpaired M_diff_ (NR vs no LV) = −118 and 95.0% CI [−196.0, −28.4], and unpaired M_diff_ (R vs no LV) = −322 and 95.0% CI [−390, −234] followed by two-sided permutation *t* test with *p* = 0.134, *p* = 0.0306, and *p* = 0.0002, respectively. *Figure Contributions*: Melina P. Bordone performed the multifactorial analysis, clustering, and statistical analyses. Presentation of data and results were discussed with Oscar S. Gershanik, M. Elena Avale, and Juan E. Ferrario.

Additionally, and in view of the wide intragroup variable responses within the miRNA-Fyn group, we performed a HCPC, including all miRNA-Fyn-injected mice as individuals, and the sum of total AIMs score for the first or second L-DOPA trial, together with their biochemical markers (see below), as variables. As a result of this analysis, two clusters were obtained (Fig. 2*A*) and the variables significantly linked with the clusters were: total AIMs score for the first and second trials and FosB levels (*p* < 0.0001, *p* < 0.0001, *p* = 0.0125, respectively). These parameters were significantly lower in one of the clusters and higher in the other, in both cases compared with the overall mean (*p* = 0.0004, *p* = 0.0013, *p* = 0.0185, respectively).

According with this analysis, the miRNA-Fyn-treated group was split into responders (R) and non-responders (NR), to the treatment, in analogy to clinical practice. We thereafter considered these two groups separately for further analysis. Although mice included in the R group exhibited similar motor deficits than NR mice in the cylinder test (Fig. 2*B*), we found that total AIMs score of the R group was lower than the other experimental groups (Fig. 2*C*,*D*). Additionally, the time course of AIMs score during the last session of the first (Fig. 2*E*) and second (Fig. 2*F*) L-DOPA trial showed that R mice developed significantly less dyskinesia during the whole sessions and this effect was persistent over time.

The sum of all AIMs scores throughout the experiment was analyzed both by null hypothesis significance testing (Fig. 2*G*) and estimation statistic (Fig. 2*H*,*H’*). The former analysis showed that the R group was statistically different from all other groups. The later, depicted as a shared control Cumming estimation plot, showed the M_diff_ and 95% CI for three comparisons against the no LV control group. In this case the R group had a M_diff_ of −322 [95.0%CI −390, −234], *p* = 0.0002, while the unpaired M_diff_ for the NR group was −118 [95.0%CI −196, −28.4], *p* = 0.0306, and for the control LV was −106 [95.0%CI −234, 5.7], *p* = 0.134. In conclusion, both statistical analyses demonstrate that the R group behaved differently than all other groups.

#### Biochemical markers and regulation of Fyn activity

##### TH immunodetection

**Figure 3.**
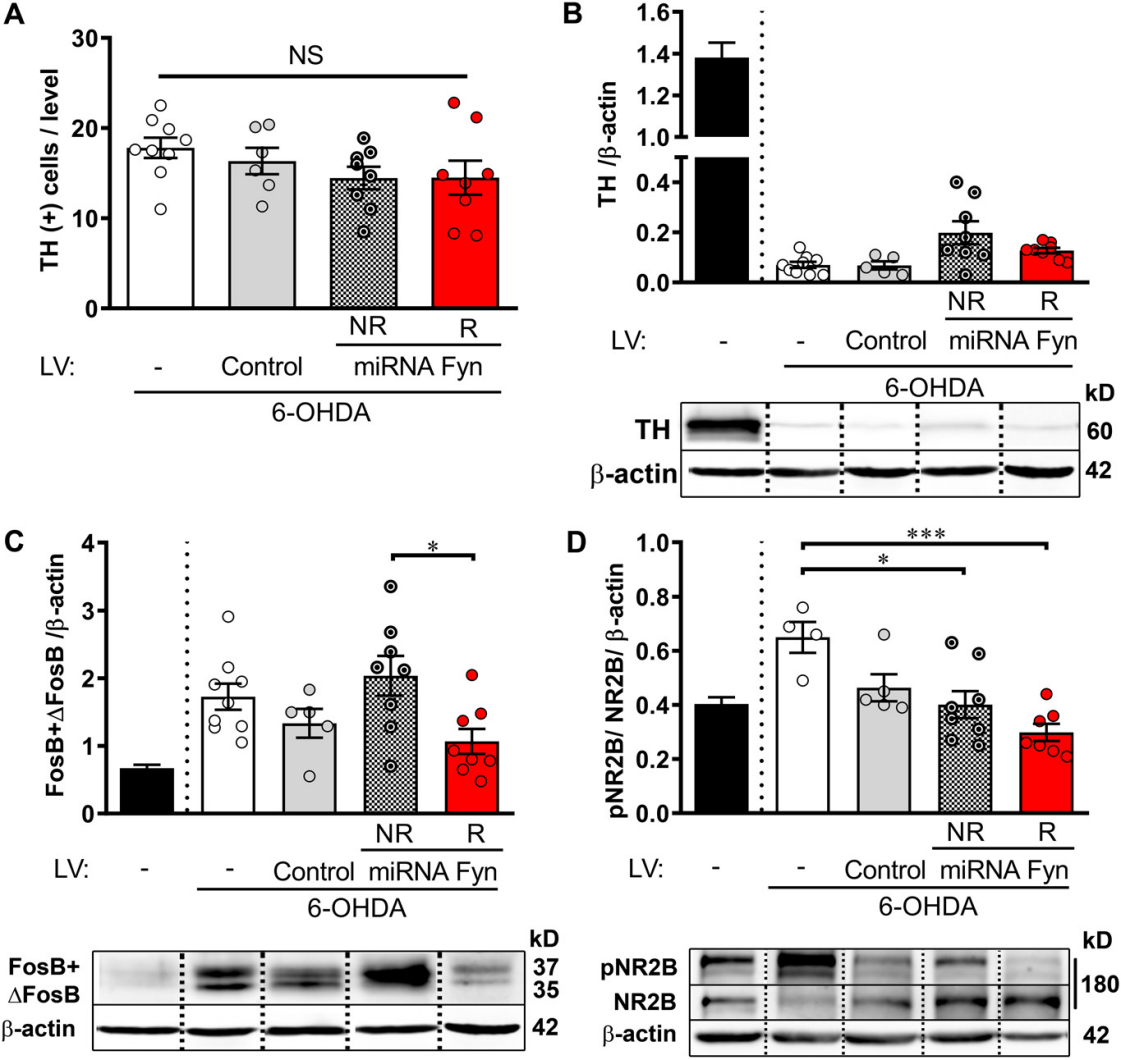
Postmortem analysis of pre-L-DOPA miRNA Fyn treatment. ***A***, TH-positive cell counting at the SNpc from immunohistochemical staining of coronal slices. One-way ANOVA (*F*_(3,27)_ = 1.344, *p* = 0.2809). ***B***, Analysis of dopamine depletion by Western blot quantification of TH/β-actin. Kruskal–Wallis test *H*_(3)_ = 11.99; *p* = 0.0074 followed by Dunn’s test (*p* = 0.0332, no LV vs NR). ***C***, FosB-ΔFosB levels relative to β-actin. One-way ANOVA (*F*_(3,26)_ = 3.583, *p* = 0.0272) and *post hoc* Tukey’s test (*p* = 0.0236, R vs NR). ***D***, Detection of neuronal Fyn activity by Western blot quantification of the phosphorylation status of NR2B in striatal homogenates. Values indicate pNR2B/NR2B/β-actin ratio. One-way ANOVA (*F*_(3,20)_ = 8.035, *p* = 0.0010) and *post hoc* Tukey’s test (*p* = 0.0114 and *p* = 0.0006, for no LV vs NR and R, respectively). In all figures, data are mean ± SEM. In ***B–D***, the black bar indicates mean ± SEM of the contralateral non-lesioned striatal samples, as a reference value and was not included in the statistical analysis. *Figure Contributions*: Juan E. Ferrario and Sara Sanz-Blasco dissected brain structures. Melina P. Bordone and Tomas Eidelman performed mesencephalic slices and immunohistochemistry of TH. Tomas Eidelman quantified TH-positive cells and Melina P. Bordone made Western blottings.

The severity of dyskinesia depends on the degree of dopamine depletion as well as dosage and timing of L-DOPA, (Bastide et al., 2015). As L-DOPA administration was equivalent in all mice, to rule out any putative difference because of the lesion extent, we performed immunodetection of TH to evaluate dopaminergic denervation both in the SNpc and in the striatum ipsilateral to the 6-OHDA-injected side. We found a similar amount of remaining TH (+) cells in the SNpc between experimental groups (Fig. 3*A*). TH protein levels in striatal homogenates assessed by Western blotting showed >85% of reduction in all groups compared with non-lesioned striata (Fig. 3*B*). Although this last determination showed more variability than cell counting in the NR group, the R mice showed very low amounts of TH (91% of reduction), compatible with high depletion of striatal dopamine. Moreover, TH content was included in the HCPC analysis showing that TH has no influence in the formation of clusters, therefore, indicating that differences observed between groups were not determined by the level of dopaminergic denervation.

##### FosB-ΔFosB protein levels

Striatal FosB-like proteins are upregulated and causally linked to LID (Andersson et al., 1999; Pavón et al., 2006; Beck et al., 2019). Therefore, we wondered whether the AIMs score achieved by each group was in consonance with striatal FosB-ΔFosB protein levels (Fig. 3*C*). In parallel with a high dyskinetic behavior, NR and control groups accumulated more than 2-fold FosB-ΔFosB in the ipsilateral side compared with the reference values determined for the contralateral side, while R mice accumulated significantly less FosB-ΔFosB than NR.

##### Fyn-mediated phosphorylation of NMDA-R-NR2B subunit (Y-1472)

The NMDA-R has been consistently implicated in LID. NMDA-R is a heterotetramer composed by two NR1 subunits and two NR2 subunits (NR2A and NR2B). Three major tyrosine residues (Y-1252, Y-1336, and Y-1472) have been identified in the NR2B C-terminus (Chen and Roche, 2007), Y-1472 being the main tyrosine phosphorylation substrate of Fyn (Roche et al., 2001; Lavezzari et al., 2003).

To determine the efficacy of miRNA-Fyn administration, and because of the limitation to detect Fyn reduction *in vivo* (see Discussion), we analyzed the phosphorylation of the NR2B subunit at Y-1472 (pNR2B) as a surrogate marker of Fyn activity. This analysis showed that pNR2B was significantly reduced in both R and NR miRNA-Fyn-injected groups compared with the no LV group (Fig. 3*D*). Yet, such reduction was more significant in the R group, suggesting a threshold of Fyn silencing required to impact on dyskinetic behavior. One-tailed Student’s *t* test also shows that pNR2B was significantly reduced in the miRNA-Fyn R group compared with the control-LV group (*t* = 2.945, df = 10; *p* = 0.0073) and reduced, but in the limit of the statistical significance, when compared with NR (*t* = 1.680, df = 13; *p* = 0.0584).

### Posttreatment schedule

#### AIMs

**Figure 4.**
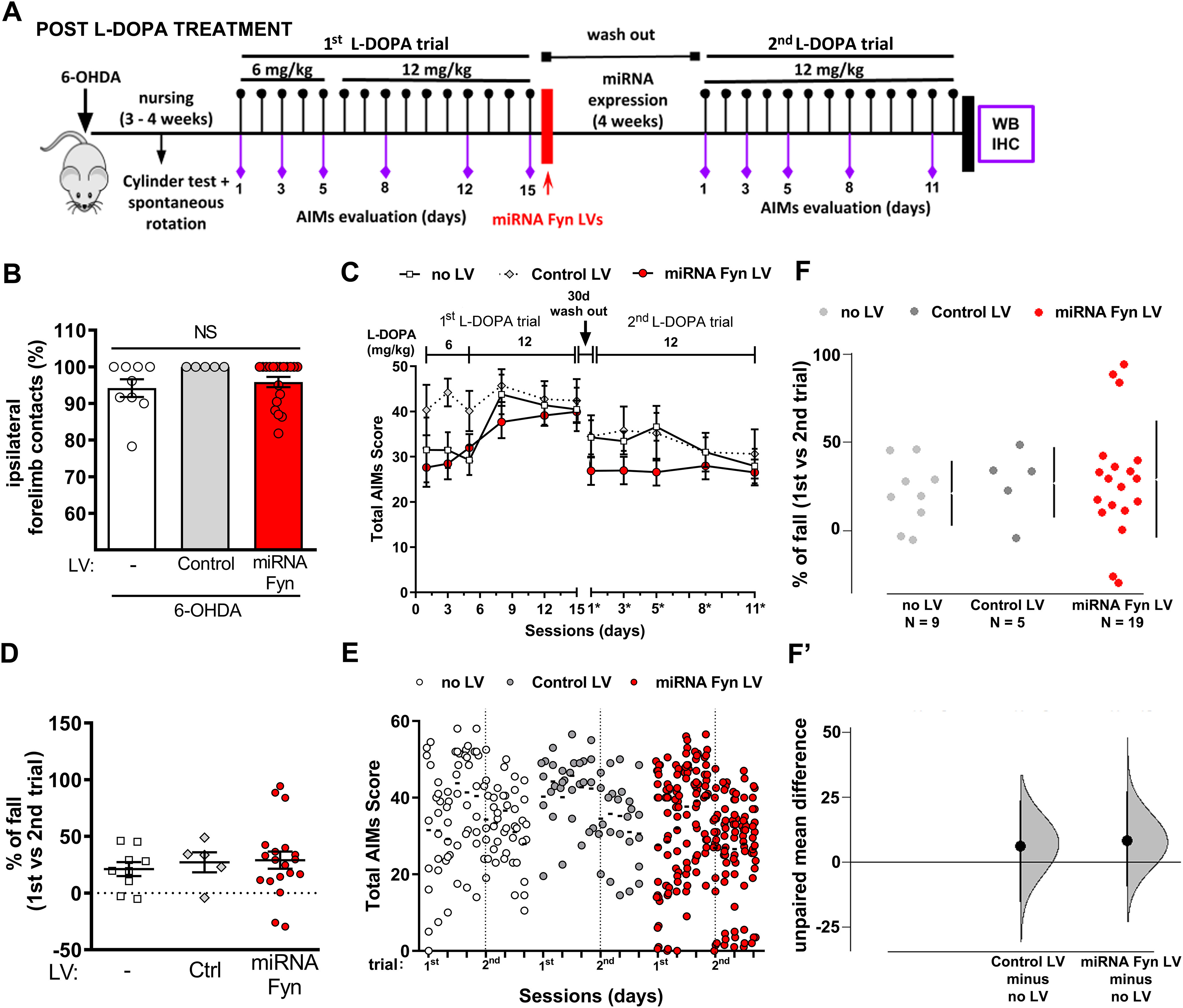
Experimental design to evaluate the reversion of LID by miRNA-Fyn treatment. ***A***, Timeline of the post-L-DOPA treatment schema: 6-OHDA lesion into the MFB followed by the first L-DOPA trial for 15 d and AIMs analysis at indicated time points; striatal miRNA LV injection, and second L-DOPA trial, AIMs evaluation, and postmortem analyses. ***B***, Cylinder test performed after 6-OHDA lesion, prior to random assignment of mice to treatment groups. Data are mean ± SEM. Kruskal–Wallis test *H*_(2)_ = 3.612; *p* = 0.1643. ***C***, Sum of values for axial dystonia, orolingual, and limb dyskinesia (total AIMs score) per day throughout the L-DOPA treatment. Experimental groups: non-injected (no LV; *n* = 9), injected with LV carrying a control (*n* = 5) or with miRNA-Fyn (*n* = 19). Data are mean ± SEM. Two-way ANOVA with repeated measures performed on data from the second L-DOPA trial (interaction: *F*_(8,120)_ = 2.520, *p* = 0.0144; time: *F*_(4,120)_ = 4.724, *p* = 0.0014; treatment: *F*_(2,30)_ = 1.089, *p* = 0.3495; subject: *F*_(30,120)_ = 38.32, *p* < 0.0001) and *post hoc* Tukey’s test did not show statistical differences between treatments. ***D***, Percentage of fall after treatments calculated for each individual mouse. Second L-DOPA trial AIM scores compared with AIM score in the first L-DOPA trial for the same group of treated mice. Data are mean ± SEM. One-way ANOVA (*F*_(2,30)_ = 0.2390 *p* = 0.7889). ***E***, Dot plot of data showing the distribution of the AIMs score within each group. The dotted line separates the first from the second L-DOPA trial. ***F***, ***F’***, Percentage of fall after treatments analyzed by estimation statistic and shown as a Cumming estimation plot. ***F***, Raw data are presented as a swarmplot and mean ± SD are represented on the right of each experimental group. ***F’***, Unpaired mean difference for two comparisons against the shared control (no LV group). The unpaired M_diff_ are plotted as bootstrap sampling distributions. Each mean difference is depicted as a dot. Each 95% CI is indicated by the ends of the vertical error bars. Unpaired M_diff_ (control LV vs no LV) = −5.99 and 95.0% CI [−16.0, 22.8] and unpaired M_diff_ (miRNA-Fyn vs no LV) = 7.89 and 95.0% CI [−9.89, 27.0] followed by two-sided permutation *t* test with *p* = 0.575 and *p* = 0.521, respectively. *Figure Contributions*: Melina P. Bordone performed 6-OHDA lesions and Ana Damianich striatal lentiviral injections. Melina P. Bordone, Ana Damianich, Tomas Eidelman, and Juan E. Ferrario made animal nursing and cylinder tests. Melina P. Bordone, Tomas Eidelman, and Sara Sanz-Blasco scored LIDs. Melina P. Bordone performed statistical analyses and Melina P. Bordone, Juan E. Ferrario, and M. Elena Avale analyzed and discussed data.

We next tested the potential of miRNA-Fyn treatment to revert already stablished dyskinesia (post-L-DOPA treatment; Fig. 4*A*). Four weeks after a 6-OHDA lesion, mice with similar motor impairment assessed by the cylinder test (Fig. 4*B*), were induced to develop dyskinesia by a first L-DOPA trial. Once finished, they randomly received an intrastriatal injection of either miRNA-Fyn LV or control LV or left non-injected (no LV). Four weeks after the LV injection a second L-DOPA trial was performed, and AIMs were registered.

During the first exposures to L-DOPA, no differences were observed in LID between groups (Fig. 4*C*). After LVs injection, control groups showed a reduction of LID of approximately five points of AIMs (10–15%), as observed in similar paradigms (Porras et al., 2012), while the miRNA-Fyn group showed a non-significant reduction of ∼15 points of AIMS (35–40%; Fig. 4*C*). Also, for each mouse, we determined the percentage of fall of their own dyskinetic score between the first and the second L-DOPA trial (% of fall), calculated using the mean AIMs score of the last three sessions of the first trial with L-DOPA and the last three sessions of the second trial. This parameter did not show statistical differences between groups neither by hypothesis testing (Fig. 4*D*) nor by estimation statistics (Fig. 4*F*,*F’*). It is remarkable the higher dispersion found within the miRNA-Fyn group (Fig. 4*D*,*F*).

**Figure 5.**
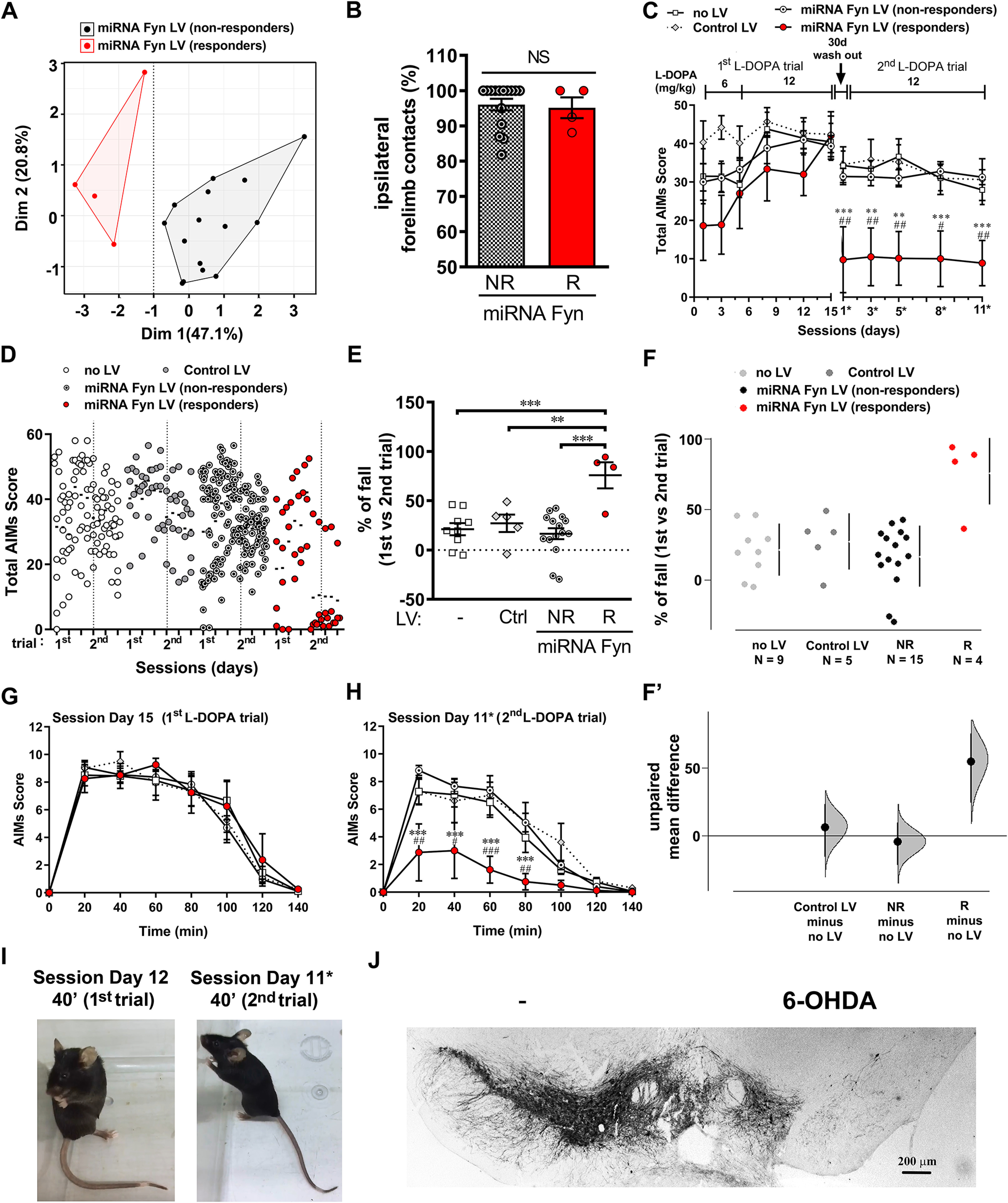
Analysis of LID reversion by miRNA-Fyn treatment. ***A***, Factor map after HCPC split miRNA Fyn-injected mice into R (*n* = 4) and NR (*n* = 15). ***B***, Cylinder test from miRNA Fyn-treated mice split into R and NR. Data are mean ± SEM. Two-tailed unpaired Student’s *t* test (*t* = 0.2521, df = 17, *p* = 0.8040). ***C***, Total AIMs score throughout the L-DOPA treatment of the experimental groups: non-injected (no LV; *n* = 9), injected with a control LV (*n* = 5) and miRNA-Fyn LV (*n* = 19). The miRNA-Fyn group was split into R (*n* = 4) and NR (*n* = 15) mice. Data are mean ± SEM. Two-way ANOVA with repeated measures performed on data from the second L-DOPA trial (interaction: *F*_(12,116)_ = 1.676, *p* = 0.0809; time: *F*_(4,116)_ = 3.007, *p* = 0.0211; treatment: *F*_(3,29)_ = 6.930, *p* = 0.0012; subject: *F*_(29,116)_ = 24.05, *p* < 0.0001), with repeated measures and *post hoc* Tukey’s test; **p < 0.01 and ***p < 0.001 versus NR and #*p* < 0.05 and *p* < 0.01 versus control LV. ***D***, Dot plot of all experimental groups. The dotted line separates the first from the second L-DOPA trial. The cloud of values of the miRNA Fyn-treated mice during the second trial (and the mean AIMs score for each day) evidenced the two subpopulations of R and NR. ***E***, Percentage of fall after treatments. Second L-DOPA trial AIM scores compared with AIM score in the first L-DOPA trial for the same group of mice. Data are mean ± SEM. One-way ANOVA (*F*_(3,29)_ = 8.625 *p* = 0.0003) and *post hoc* Tukey’s test; **p < 0.01 and ***p < 0.001. ***F***, ***F’***, Percentage of fall after treatments analyzed by estimation statistic and shown as a Cumming estimation plot. ***F***, The raw data are presented as a swarmplot and mean ± SD are represented on the right of each experimental group. ***F’***, Unpaired mean difference for three comparisons against the shared control (no LV group). The unpaired M_diff_ are plotted as bootstrap sampling distributions. Each mean difference is depicted as a dot. Each 95% CI is indicated by the ends of the vertical error bars. Unpaired M_diff_ (control LV vs no LV) = −5.99 and 95.0% CI [−16.0, 22.8], unpaired M_diff_ (NR vs no LV) = −4.59 and 95.0% CI [−20.7, 10.6], and unpaired M_diff_ (R vs no LV) = 54.7 and 95.0% CI [20.9, 73.7] followed by two-sided permutation *t* test with *p* = 0.575, *p* = 0.603, and *p* = 0.0026, respectively. ***G***, ***H***, AIMs score during session day 15 (first trial with L-DOPA; ***G***) and session day 11* (second trial with L-DOPA; ***H***). Data are mean ± SEM. ***G***, Two-way repeated measures ANOVA (interaction: *F*_(21,210)_ = 0.6627, *p* = 0.8663; time: *F*_(7,210)_ = 130.2, *p* < 0.0001; treatment: *F*_(3,30)_ = 0.05,039, *p* = 0.9848; subject: *F*_(30,210)_ = 5.940, *p* < 0.0001. ***H***, Two-way repeated measures ANOVA (interaction: *F*_(21,203)_ = 3.011, *p* < 0.0001; time: *F*_(7,203)_ = 73.81, *p* < 0.0001; treatment: *F*_(3,29)_ = 5.764, *p* = 0.0032; subject: *F*_(29,203)_ = 4.457, *p* < 0.0001), both followed by *post hoc* Tukey’s test; ***p < 0.001 versus NR; #*p* < 0.05, ##*p* < 0.01, and ###*p* <0.001 versus control LV. ***I***, Video captures of a mouse subjected to post-L-DOPA miRNA Fyn treatment at two representative days from both trials with L-DOPA before (Movie 1, left panel) or after the miRNA-Fyn treatment, showing full recovery after Fyn silencing (Movie 2, right panel). Movie 1 was registered from a 6-OHDA-lesioned mouse developing high dyskinesia 40 min after challenged with 12 mg/kg of L-DOPA. Movie 2 was registered from the same mouse after having received the intrastriatal injection of lentiviral miRNA-Fyn, showing no dyskinesia 40 min after challenged with 12 mg/kg of L-DOPA. ***J***, Immunodetection of TH in the SNpc corresponding to the mouse shown in the movies. *Figure Contributions*: Melina P. Bordone performed the multifactorial analysis, clustering, statistical analyses and illustrative movies and photograph. Presentation of data and results were discussed with Oscar S. Gershanik, M. Elena Avale, and Juan E. Ferrario.

The scatter dot plots showed that some mice strongly reduced their previous AIMs score during the second trial (Fig. 4*D–F*). Hence, a HCPC analysis was performed on the miRNA-Fyn LV-injected group using the biochemical variables and the percentage of fall on LID. The HCPC analysis showed two groups, that were again split into R and NR. The variables significantly linked with the clusters were: the % of fall, FosB, and pNR2B levels (*p* = 0.0002, *p* = 0.0006, and *p* = 0.0058, respectively). These parameters were significantly lower in one of the clusters and higher in the other, in both cases compared with the overall mean (*p* = 0.0014; *p* = 0.0026; and *p* = 0.0100, respectively). However, in this experimental paradigm the R group only represented the 21% (4/19) of the miRNA-Fyn-treated mice (Fig. 5*A*), suggesting that reversion of already stablished LID is less effective that prevention, at least, in this experimental design and conditions. Both groups presented a similar profile in the cylinder test (Fig. 5*B*) and in total AIMs score before miRNA-Fyn treatment (Fig. 5*C*), while the R group developed a total AIMs score significantly lower during the second L-DOPA trial (Fig. 5*C*), which is observable when plotting the dot distribution (Fig. 5*D*). The R group showed a statistically significant decrease in LID compared with the other experimental groups (Fig. 5*E*). Also, the estimation based on CIs for three comparisons against the shared control (no LV group) showed unpaired M_diff_ for control LV of −5.99 [95.0% CI −16.0, 22.8] *p* = 0.575, and for NR of −4.59 [95.0% CI −20.7, 10.6] *p* = 0.603 while the unpaired M_diff_ for R was 54.7 [95.0% CI 20.9, 73.7] *p* = 0.0026 (Fig. 5*F*,*F’*).

Movie 1. Representative movie of a typical 6-OHDA-lesioned mouse developing high dyskinesias 40 min after challenged with 12 mg/kg of L-DOPA. 10.1523/ENEURO.0559-20.2021.video.1

Movie 2. Representative movie of the same mouse after having received the intrastriatal injection of lentiviral miRNA-Fyn, showing no dyskinesias 40 min after challenged with 12 mg/kg of L-DOPA. 10.1523/ENEURO.0559-20.2021.video.2

The analysis of the dyskinetic profile during a representative session showed no differences between groups during the first trial with L-DOPA (Fig. 5*G*) but after the miRNA-Fyn treatment, R mice exhibited a significant decrease of >50% of LID compared with control LV and NR (Fig. 5*H–I*; see also movies before and after miRNA-Fyn injection, Movies 1, 2) in a R mouse exhibiting dramatic nigral dopaminergic loss (Fig. 5*J*).

### Biochemical markers

**Figure 6.**
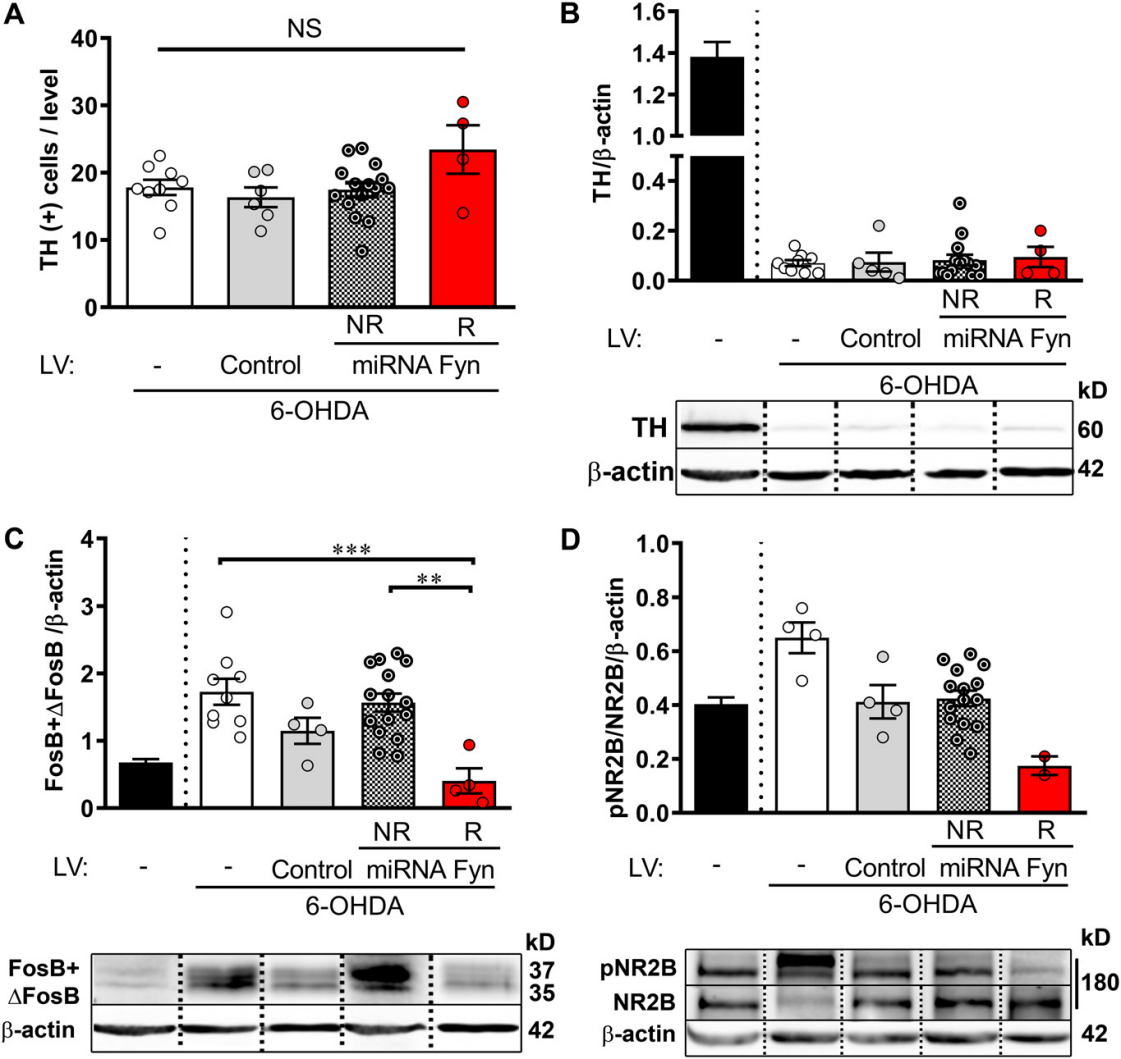
Postmortem analysis of post-L-DOPA miRNA Fyn treatment. ***A***, TH-positive cell counting at the SNpc from immunohistochemical staining of coronal slices. One-way ANOVA (*F*_(3,30)_ = 2.596, *p* = 0.0708). ***B***, Analysis of dopamine depletion by Western blot quantification of TH/β-actin. Kruskal–Wallis test *H*_(3)_ = 0.6519; *p* = 0.8844. ***C***, FosB-ΔFosB levels relative to β-actin. One-way ANOVA (*F*_(3,28)_ = 7.174, *p* = 0.0010) and *post hoc* Tukey’s test (*p* = 0.0010 for no LV vs R and *p* = 0. 0020 for R vs NR). ***D***, Detection of neuronal Fyn activity by Western blot quantification of the phosphorylation status of NR2B in striatal homogenates. Values indicate pNR2B/NR2B/β-actin ratio. No statistical analysis was applied because of two missing values in the R group. In all figures, data are mean ± SEM. In ***B–D***, the black bar indicates mean ± SEM of the contralateral non-lesioned striatal samples, as a reference value and was not included in the statistical analysis. *Figure Contributions*: Juan E. Ferrario and Sara Sanz-Blasco dissected brain structures. Melina P. Bordone and Tomas Eidelman performed mesencephalic slices and immunohistochemistry of TH. Tomas Eidelman quantified TH-positive cells and Melina P. Bordone made Western blottings.

Immunodetection of TH demonstrated a similar degree of dopaminergic degeneration between experimental groups, both at TH (+) cell counting in the SNpc (Fig. 6*A*) or striatal TH protein levels (Fig. 6*B*).

All 6-OHDA-lesioned mice challenged with L-DOPA showed increased FosB-ΔFosB compared with the contralateral side as expected with the high dyskinetic score, however, the R group significantly accumulated less FosB-ΔFosB than other groups, compatible with the low dyskinetic profile developed after the miRNA-Fyn treatment (Fig. 6*C*).

Finally, analysis of Fyn-mediated phosphorylation of NR2B subunit (Y-1472) suggests that responders to the miRNA-Fyn LV have lower NR2B phosphorylation, but these values were not included into a statistical analysis because of two missing values in this small subgroup (Fig. 6*D*).

## Discussion

In this study, we demonstrate that local silencing of the Src kinase Fyn ameliorates LID in a mouse model of PD. We designed a miRNA against Fyn that was injected into the ipsilateral striatum of dyskinetic mice. Results presented here validate Fyn as a novel target to reduce LID and set the grounds for the development of new therapies.

We compared the preventive (pre-L-DOPA miRNA-Fyn treatment) versus restorative (post-L-DOPA miRNA-Fyn treatment) effects of miRNA-Fyn injection. The preventive therapeutic effect of all miRNA-Fyn-treated mice together significantly reduces LID in the order of 40% and 57%, comparing to the control LV or the no LV group, respectively (Fig. 1*G*). This reduction is comparable to that observed with amantadine (Cenci and Lundblad, 2007) or the knock-down of PSD-95 (Porras et al., 2012). In our experimental approach, it is noteworthy that in both pre-L-DOPA and post-L-DOPA schedule, several animals did not respond to miRNA-Fyn treatment. LID is a very variable phenotype both in humans and animal models, yielding wide data dispersion. This feature makes it necessary to perform a meticulous data analysis as the behavior of a single animal could make the difference between the significance or not of any effect. Sometimes, this issue is solved by increasing the number of replicas, in opposition to ethical recommendations. Here, we explored different alternatives to analyze experimental dyskinetic behaviors. On one hand, as a complement to the usually used null-hypothesis significance testing, we analyzed data with estimation statistic, which consider data dispersion for each group (represented in a CI and scatter plot of data) and estimate the potential differences between them (Calin-Jageman and Cumming, 2019). On the other hand, we took into consideration that the intrinsic variability, in addition to experimental factors (as dopaminergic denervation) or treatment efficiency (here as LV injection), makes necessary to perform meticulous postmortem analysis. We therefore used a refined algorithm of data analysis, the HCPC. Nearly 50% of the miRNA-Fyn-injected mice prior to L-DOPA administration showed a significant and robust reduction of LID. Those mice that responded to the miRNA-Fyn treatment also showed a significant reduction in FosB-ΔFosB protein levels and decreased phosphorylation of NR2B as a surrogate marker of Fyn activity, both compatible with the behavioral data. On the other hand, expression of miRNA-Fyn once LID was consolidated (post-L-DOPA) was less efficient in our experimental conditions, although 21% of treated mice responded to treatment. In humans, the existence of “response rates” is a common clinical feature in any clinical drug trial and in clinical practice (Atkinson et al., 2019). As an example, in a large multicenter study, it was shown that the efficacy of amantadine in PD patients suffering from dyskinesia was only 64% (Sawada et al., 2010). The response of patients to medical treatment is influenced by a variety of environmental, pathologic, physiological, and genetic factors, speculated but not determined. Detection of R and NR animals by statistical methods has started to be used in experimental physiology and it is suggested by ethic committees to reduce the number of experimental animals aiming to adjust to the 3Rs principle.

In conclusion, we found that estimation statistic based on CIs is strong to complement and support results also analyzed by null hypothesis testing. In addition, the multivariate analysis is a powerful tool for identifying sub-groups of experimental subjects.

Overall, the results observed in the R groups are encouraging to further investigation and technical refinements. The differential response between pre-L-DOPA and post-L-DOPA treatment schedules suggests that the plastic (maladaptative) rearrangement taking place in the striatum after L-DOPA induction of dyskinesia are consolidated and difficult to revert. Remarkably, we observed a similar result, previously, when treating dyskinetic mice with the non-specific Fyn antagonist, saracatinib, which, in our experimental conditions, was able to prevent but not to revert LID (Sanz-Blasco et al., 2018).

It would be of great interest to determine how Fyn silencing may affect the beneficial effect of L-DOPA. Unfortunately, we were unable to accurately measure the improvement of motor function in the cylinder test, probably because the doses of L-DOPA that we used to induce dyskinesia masked the exploration in the cylinder precluding the use of the ipsilateral paw. It was also not possible to determine it at the end of the dyskinetic period, because duration of LID was very variable for each mouse and experimental group (as seen in Figs. 2*E*,*F*, 5*H*), making results inconsistent. Despite the absence of quantitative determination, we observed that those mice treated with miRNA-Fyn, freely and actively explored their cages during the pharmacological window of effect of L-DOPA. An example of this can be seen in Movie 2. Based on this observation, we can safely affirm that in those miRNA-Fyn-treated mice with a reduced level of LID, it is still capable of reversing the typical lethargy of 6-OHDA-lesioned mice. A fine analysis of this crucial point is necessary on subsequent work.

Determination of Fyn reduction by Western blotting in cultured neuronal cells demonstrated that the miRNA-Fyn (miRNA 691) chosen for this study was able to reduce Fyn protein levels by 50% in most of the replicas (Fig. 1*C*). A previous report showed that similar rates of *in vitro* Fyn silencing were enough to evidence an *in vivo* effect (Phamluong et al., 2017). However, both in that study, as in our hands, Fyn reduction was not detectable in brain homogenates. This can be because of the fact that Fyn is highly expressed in glial cells, and as we used a neuron-specific (synapsin) promoter for miRNA-Fyn expression, neuronal Fyn reduction might have been masked by glial Fyn. To overcome this problem, we determined Fyn-specific Y-1472 phosphorylation of the NR2B subunit as an indirect measure of Fyn knock-down in neurons. Responder groups showed a concomitant reduction in pNR2B/NR2B ratio and LID. Considering that Fyn is not the only regulator of Y-1472-pNR2B (Ali and Salter, 2001), this result suggests that Fyn silencing was successfully achieved in this group. On the other hand, NR groups showed intermediate values for pNR2B/NR2B suggesting that a weaker silencing by the miRNA-Fyn was not enough to reduce LID. It is tempting to hypothesize the existence of a threshold influencing the outcome of the miRNA-Fyn treatment, because of several factors, such as kinetics of NR2B phosphorylation/dephosphorylation after L-DOPA administration, or compensation of other pNR2B modulators on Fyn downregulation. However, the reduced severity of the dyskinetic behavior after miRNA-Fyn expression appears to be a reliable measure of therapeutic benefit.

Precision is the hallmark of next generation therapy. Future pharmacological strategies based on DNA or RNA therapy would complement classic pharmacology in many diseases still unsatisfactorily treated. Precision given by gene or RNA therapy reduces physiological side effects because of their intrinsic target specificity and local effects. Particularly, in neurodegenerative disease, if ubiquitous molecules need to be targeted, it is advisable to restrict its blockade to the affected brain nuclei, to avoid side effects because of their overall reduction. To this end, viral vectors are promising tools which are already being tested (Naldini et al., 2016). Indeed, clinical trials using gene therapy for PD are already underway (Hitti et al., 2019), and one in particular using LV (Palfi et al., 2014) that has shown safety, tolerability, and sustained beneficial effects in treated patients (Palfi et al., 2018).

To attain the desired therapeutic effect, in addition to targeting the intended molecule, it is necessary to focalize a restricted brain region and the precise cell type. In this work we sought to cover the striatum, especially the dorsolateral region, that has been mostly related with LID. To achieve a tailored, specific effect, we targeted neurons, not glia; however, unraveling the role of Fyn in each subpopulation of MSNs is of the utmost importance. Current evidence indicates that a subpopulation of FosB-ΔFosB actively expressing striatal neurons (many dMSN, a handful parvalbumin-positive interneurons, and a small number of MSNs expressing D2-R) are also implicated in LID development (Girasole et al., 2018). In addition, optogenetic stimulation of the direct pathway terminals demonstrated the ability of these neurons per se to develop “light”-induced dyskinesia in 6-OHDA-lesioned mice, indistinguishable from standard LID (Keifman et al., 2019). The role of D2-expressing indirect MSN (iMSN) in LID is still controversial and its specific chemogenetic stimulation reduces dyskinesia (Alcacer et al., 2017), it is then tempting to speculate that stimulation of iMSN may have an opposite functional role than on dMSN and therefore the effect of silencing Fyn in these two neuronal types could not be necessarily additive, contrariwise, they could be opposite. The cellular complexity underlying LID development and maintenance rises the need to further dissect the role of each involved cellular type, to increase the precision of therapeutic approaches.

NMDA-R as a target for LID reduction is unquestionable effective, but side effects are still difficult to control. The rationale presented in this article attempts to reduce NMDA-R activity, and therefore LID, through an approach directed at interfering with the underlying biological mechanism that will ultimately affect the physiological function. This line of reasoning led to the development of the NR2B antagonist CP-101606, but clinical trials were aborted because of side effects (Nash et al., 2004; Nutt et al., 2008; Kong et al., 2015). The potential development of another, and maybe more specific drug as antagonist, however, will not overcome the risk of modifying biological functions in other areas of the brain, as mentioned above. As Fyn is a key regulator of the NR2B subunit, targeting *Fyn* mRNA at the specific striatal neurons should reduce Fyn and NMDA-R activity and, consequently, LID.

Results presented here are in line with this reasoning and strongly validate Fyn as a novel target against LID, complementing previous work (for review, see Angelopoulou et al., 2021). The interest in the target, together with the precision achieved by gene and RNA therapies, represents a powerful combination to be further explored and translated in the near future as an alternative therapeutic option for the management of LID in PD.

## Synthesis

Reviewing Editor: Alexxai Kravitz, Washington University in St. Louis Decisions are customarily a result of the Reviewing Editor and the peer reviewers coming together and discussing their recommendations until a consensus is reached. When revisions are invited, a fact-based synthesis statement explaining their decision and outlining what is needed to prepare a revision will be listed below. The following reviewer(s) agreed to reveal their identity: NONE. Note: If this manuscript was transferred from JNeurosci and a decision was made to accept the manuscript without peer review, a brief statement to this effect will instead be what is listed below.

I comment you on an excellent paper and being very responsive to comments from reviewers. I have read the paper, the prior reviews, and your rebuttal and I plan to make a decision without sending it back to them for additional review.

I have only one minor comment to address, in that I agree with Reviewer 2 that the “case study” mouse in Figure 7 is not useful to show. As the authors mention, the high degree of variance in both LIDs and their modulation by treatment, showing a single mouse like this confuses rather than clarifies the results. For example, we have all seen “responses” in saline-treated control mice, which only make sense to look at in the context of larger groups of animals.

That said, I agree that the videos they show are impressive, and it is nice to see the video demonstrations of behavioral effects. I therefore prefer if the authors remove Figure 7 but reference the individual example supplemental movies as an example of a strong responder.
